# Spatiotemporal dynamics of HIV-1 CRF63_02A6 sub-epidemic

**DOI:** 10.3389/fmicb.2022.946787

**Published:** 2022-08-31

**Authors:** Mariya V. Sivay, Lada V. Maksimenko, Irina P. Osipova, Anastasiya A. Nefedova, Mariya P. Gashnikova, Dariya P. Zyryanova, Vasiliy E. Ekushov, Alexei V. Totmenin, Tatyana M. Nalimova, Vladimir V. Ivlev, Dmitriy V. Kapustin, Larisa L. Pozdnyakova, Sergey E. Skudarnov, Tatyana S. Ostapova, Svetlana V. Yaschenko, Olga I. Nazarova, Aleksander S. Chernov, Tatyana N. Ismailova, Rinat A. Maksutov, Natalya M. Gashnikova

**Affiliations:** ^1^Department of Retroviruses, State Research Center of Virology and Biotechnology “Vector”, Koltsovo, Russia; ^2^City Infectious Clinical Hospital #1, Novosibirsk, Russia; ^3^Krasnoyarsk Regional Center for Prevention and Control of AIDS, Krasnoyarsk, Russia; ^4^Omsk City Center of Prevention and Control of AIDS and Other Infectious Diseases, Omsk, Russia; ^5^Tomsk Regional Center for Prevention and Control of AIDS and Other Infectious Diseases, Tomsk, Russia

**Keywords:** HIV-1, CRF63_02A6 HIV-1, Russia, Central Asia, spatiotemporal dynamics, phylogenetic analysis, HIV-1 epidemic

## Abstract

HIV-1 epidemic in Russia is one of the fastest growing in the world reaching 1.14 million people living with HIV-1 (PLWH) in 2021. Since mid-1990s, the HIV-1 epidemic in Russia has started to grow substantially due to the multiple HIV-1 outbreaks among persons who inject drugs (PWID) leading to expansion of the HIV-1 sub-subtype A6 (former Soviet Union (FSU) subtype A). In 2006, a local HIV-1 sub-epidemic caused by the distribution of novel genetic lineage CRF63_02A6 was identified in Siberia. In this study, we used a comprehensive dataset of CRF63_02A6 *pol* gene sequences to investigate the spatiotemporal dynamic of the HIV-1 CRF63_02A6 sub-epidemic. This study includes all the available CRF63_02A6 HIV-1 *pol* gene sequences from Los Alamos National Laboratory (LANL) HIV Sequence Database. The HIV-1 subtypes of those sequences were conferred using phylogenetic analysis, and two automated HIV-1 subtyping tools Stanford HIVdb Program and COMET. Ancestral state reconstruction and origin date were estimated using Nextstrain. Evolutionary rate and phylodynamic analysis were estimated using BEAST v 1.10.4. CRF63_02A6 was assigned for 872 *pol* gene sequences using phylogenetic analysis approach. Predominant number (n = 832; 95.4%) of those sequences were from Russia; the remaining 40 (4.6%) sequences were from countries of Central Asia. Out of 872 CRF63_02A6 sequences, the corresponding genetic variant was assigned for 75.7 and 79.8% of sequences by Stanford and COMET subtyping tools, respectively. Dated phylogenetic analysis of the CRF63_02A6 sequences showed that the virus most likely originated in Novosibirsk, Russia, in 2005. Over the last two decades CRF63_02A6 has been widely distributed across Russia and has been sporadically detected in countries of Central Asia. Introduction of new genetic variant into mature sub-subtype A6 and CRF02_AG_FSU_ epidemics could promote the increase of viral genetic diversity and emergence of new recombinant forms. Further HIV-1 studies are needed due to a continuing rapid virus distribution. Also, the implementation of HIV-1 prevention programs is required to reduce HIV-1 transmission. This study also highlights the discrepancies in HIV-1 subtyping approaches. The reference lists of HIV-1 sequences implemented in widely used HIV-1 automated subtyping tools need to be updated to provide reliable results.

## Introduction

**Figure 1:**
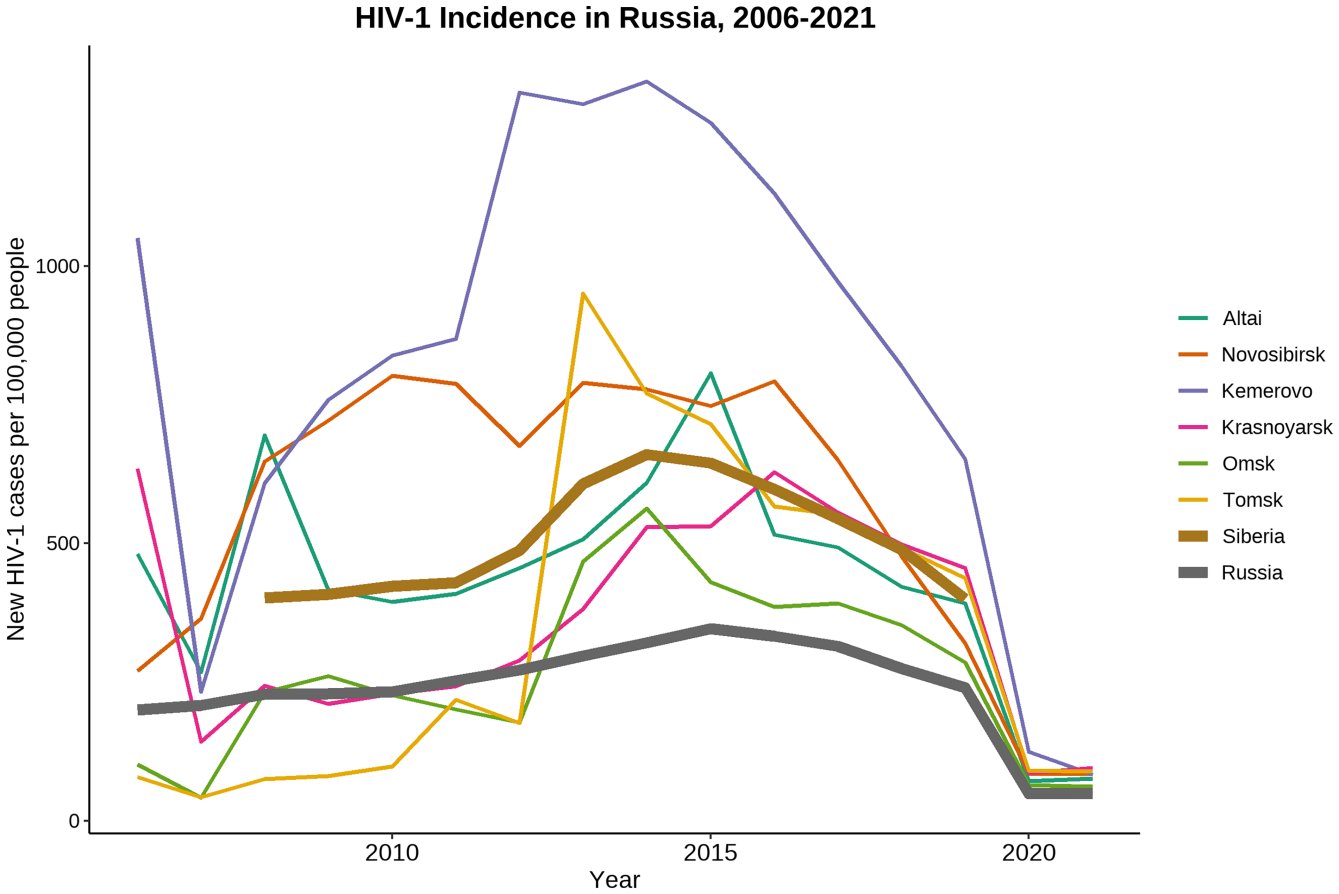
Dynamics of HIV-1 incidence in Russia from 2006 to 2021. Data were adopted from the Central Scientific Research Institute of Epidemiology of Rospotrebnadzor, Russian Federal AIDS Center, Moscow, Russia. Incidence data collected in 2019–2021 was most likely biased due to the COVID-19 world pandemic and represented the under-registered new HIV-1 cases.

Russia, as well as other countries of Eastern Europe and Central Asia, has one of the fastest growing HIV-1 epidemics in the world (Frank et al., 2019). In 2021, 1.14 million people are living with HIV-1 (PLWH) in Russia (Central Research Institute of Epidemiology, Federal Service on Customers’ Rights Protection and Human Well-being Surveillance, 2021)^1^. Russia has a diverse HIV-1 epidemic with unique patterns shaped by various socio-economical, geographical, and cultural factors influencing the development of local HIV-1 sub-epidemics within the country (Bobkova, 2013). Since mid-1990s, the HIV-1 epidemic in Russia has started to grow explosively due to the multiple HIV-1 outbreaks occurred among PWID leading to expansion of the HIV-1 sub-subtype A6, also known as former Soviet Union (FSU) subtype A (FSU-A). This genetic variant predominates in most of the Russian territories ever since (Bobkov et al., 2004; Thomson et al., 2009; Diez-Fuertes et al., 2015; Aibekova et al., 2018). In late-1990s, HIV-1 sub-epidemic among different high-risk groups was detected on the territories of the Russian Fast East (Kazennova et al., 2014; Kotova et al., 2019) and European part of Russia (Liitsola et al., 2000; Lebedev et al., 2020; Ozhmegova et al., 2020). Currently, along with the predominant sub-subtype A6, subtypes B, C, and CRF03_AB have been constantly identified (Karamov et al., 2018; Pasechnik and Blokh, 2018; Kotova et al., 2019; Safina et al., 2022). In 2006, a local HIV-1 sub-epidemic was caused by the distribution of the novel genetic lineage CRF63_02A6 in Siberia (Baryshev et al., 2012, 2014). The detailed analysis of CRF63_02A6 genome structure revealed that this viral variant originated due to recombination between Russian sub-subtype A6 and CRF02_AG_FSU_ (Central Asian lineage of the CRF02_AG; Carr et al., 2005; Baryshev et al., 2014). Several outbreaks of the CRF63_02A6 were detected in different Siberian regions between 2007 and 2014. This genetic variant has dominated in Siberia since that time (Gashnikova et al., 2015, 2017; Ulyanova et al., 2019; Kazennova et al., 2020; Maksimenko et al., 2020). The number of new HIV-1 cases has been gradually rising in Russia and has peaked in 2014, followed by the decline stage (Figure 1). At the same time, HIV-1 prevalence in Siberia was significantly higher than the country average.

Initial HIV-1 epidemic in Russia occurred mainly among PWID and their sexual partners. But since 2016, the number of people infected through the heterosexual contacts exceeded the PWID cases and the epidemic has transmitted to the general population (Bobkov et al., 1998; Bobkova, 2013; Kazennova et al., 2014; Kotova et al., 2019; Schlösser et al., 2020).

Since 2008, our laboratory has extensively collected HIV-1 sequences in Siberian regions. Beginning in 2016, the HIV-1 surveillance studies have been conducted in the Central Asia countries as a part of the national HIV-1 drug resistance surveillance program. In this study, we used a comprehensive dataset of 872 *pol* gene HIV CRF63_02A6 sequences from Russia and countries of Central Asia to investigate the spatiotemporal dynamic of HIV-1 CRF63_02A6 sub-epidemic.

## Materials and methods

### Study population and HIV-1 sequence dataset

This study uses HIV-1partial *pol* gene sequences (1,047 base pairs, HXB2 #K03455 positions 2,252–3,299, n = 587) from our previous surveillance studies (Baryshev et al., 2012; Gashnikova et al., 2015, 2017; Ulyanova et al., 2019; Maksimenko et al., 2020; Sivay et al., 2021). Blood samples were collected from 2009 to 2020 from PWLH, both ART-naïve and therapy experienced, and were used for routine antiretroviral drug resistance testing. Samples were collected in six regions of Russia (Novosibirsk, Altai, Tomsk, Omsk, Krasnoyarsk, and Kemerovo). Further manipulations with the RNA/DNA samples as well as the population sequencing procedure were previously described (Sivay et al., 2021). Additional *pol* gene sequences were retrieved from Los Alamos National Laboratory (LANL) HIV Sequence Database (Los Alamos National Laboratory HIV Sequence Database, 2022a)^2^; all sequences classified as CRF63_02A6, CRF63_01A, CRF02A6, CRF02_AG, and “other” subtypes from FSU countries were included. A limited number of CRF02_AG sequences from African and European countries were also included in the dataset; those sequences were selected randomly.

### HIV-1 subtyping

HIV-1 subtypes were identified by phylogenetic analysis. Study sequences were combined with the 2020 list of LANL HIV-1 subtype reference sequences (including all pure subtypes and CRFs) and the further phylogenetic analysis was performed with IQ-Tree (Nguyen et al., 2015) using maximum likelihood method and GRT + G4 + I substitution model and Shimodaira-Hasegawa approximate likelihood-ratio test with 1,000 replicates. HIV-1 subtype was assigned if study sequence falls into monophyletic clade with the corresponding subtype reference sequence with the branch support value ≥90. HIV-1 inter- and intra-subtype recombinants were identified using GARD (Kosakovsky Pond et al., 2006). In addition to the phylogenetic analysis, we applied the two widely used automated HIV-1 subtyping tools -- Stanford HIVdb program (Liu and Shafer, 2006) and COMET (Struck et al., 2014). The other widely used HIV-1 automated subtyping tool – REGA – was not used in this study; list of reference sequences that is used in REGA is outdated and does not include CRF63_02A6 sequences.

### HIV-1 phylogenetic and transmission cluster analyses

Maximum likelihood phylogenetic tree was constructed using IQ-Tree under GTR + 4G + I substitution model as selected by jModelTest2 (Darriba et al., 2012). Transmission clusters were identified using Cluster Picker v1.2.3 (Ragonnet-Cronin et al., 2013) with the branch support threshold of 90 and maximum genetic distance threshold of 0.03.

### Estimation of HIV-1 CRF63_02A6 origin time and location

To estimate ancestral state reconstruction and identify time to the most recent common ancestor (tMRCA) of the CRF63_02A6, a local instance of Nextstrain (Hadfield et al., 2018) was used. This is a recently developed software for real-time tracking of pathogens evolution such as COVID-19. To estimate the evolutionary rate and examine demographic history of the HIV-1 CRF63_02A6, a Bayesian Markov Chain Monte Carlo (MCMC) approach incorporated into BEAST v1.10.4 (Suchard et al., 2018) was used. To reduce computational complexity, a subsampling step was introduced, so that only a single sequence per transmission cluster was included in the further spatiotemporal analysis. The GTR + 4G substitution model, the lognormal uncorrelated relaxed clock model, and the Bayesian skyline coalescent model were used. Two independent MCMC runs of 15×10^7^ simulations were performed. Convergence of the MCMC results was examined in Tracer v1.7.1 (Rambaut et al., 2018) with effective sampling size (ESS) >200 for all parameters considered acceptable. The maximum clade credibility (MCC) tree was generated using TreeAnnotator v1.10.4 and visualized in FigTree v1.4.3 (Rambaut, 2009). We also estimated tMRCA and investigated geographic distribution of the CRF63_02A6 using BEAST v1.10.4 and compared it with the results obtained by the Nextstrain. Migration events of the HIV-1 CRF63_02A6 were reconstructed using the discrete Bayesian phylogeographic approach combined with the Bayesian Stochastic Search Variable Selection (BSSVS). Geographic location of sequences was assigned as a sampling city; if sampling city data was unavailable, then the country’s largest city location was used. Viral migration routes were summarized using SPREAD3 v0.9.7.1 (Bielejec et al., 2016).

## Results

### HIV-1 sequences dataset

A total of 2,637 HIV-1 *pol* gene sequences were included in the initial dataset using the described criteria and were used in phylogenetic analysis. Out of those sequences, 872 sequences were identified as HIV-1 CRF63_02A6, 1,157 sequences were identified as CRF02_AG_FSU_, 412 sequences were from CRF02_AG_AF/EU_ clade, and 196 sequences were classified as unique recombinant form (URF) (Supplementary Figure S1). We compare HIV-1 subtyping results obtained from the phylogenetic analysis with those from Stanford and COMET automated HIV-1 subtyping tools. Out of the sequences identified as CRF63_02A6 by phylogenetic analysis, subtyping results by Stanford and COMET were concordant for 660 (75.7%) and 696 (79.8%) sequences, respectively. Moreover, out of the full sequence dataset, COMET classified 1,092 (41.4%) sequences as CRF63_02A6; 37.5% (409/1,092) of those sequences were classified as CRF_02AG_FSU_ by phylogenetic analysis. The detailed information about subtyping results is presented in Supplementary Figure S2.

### Phylogenetic and transmission cluster analyses

**Figure 2:**
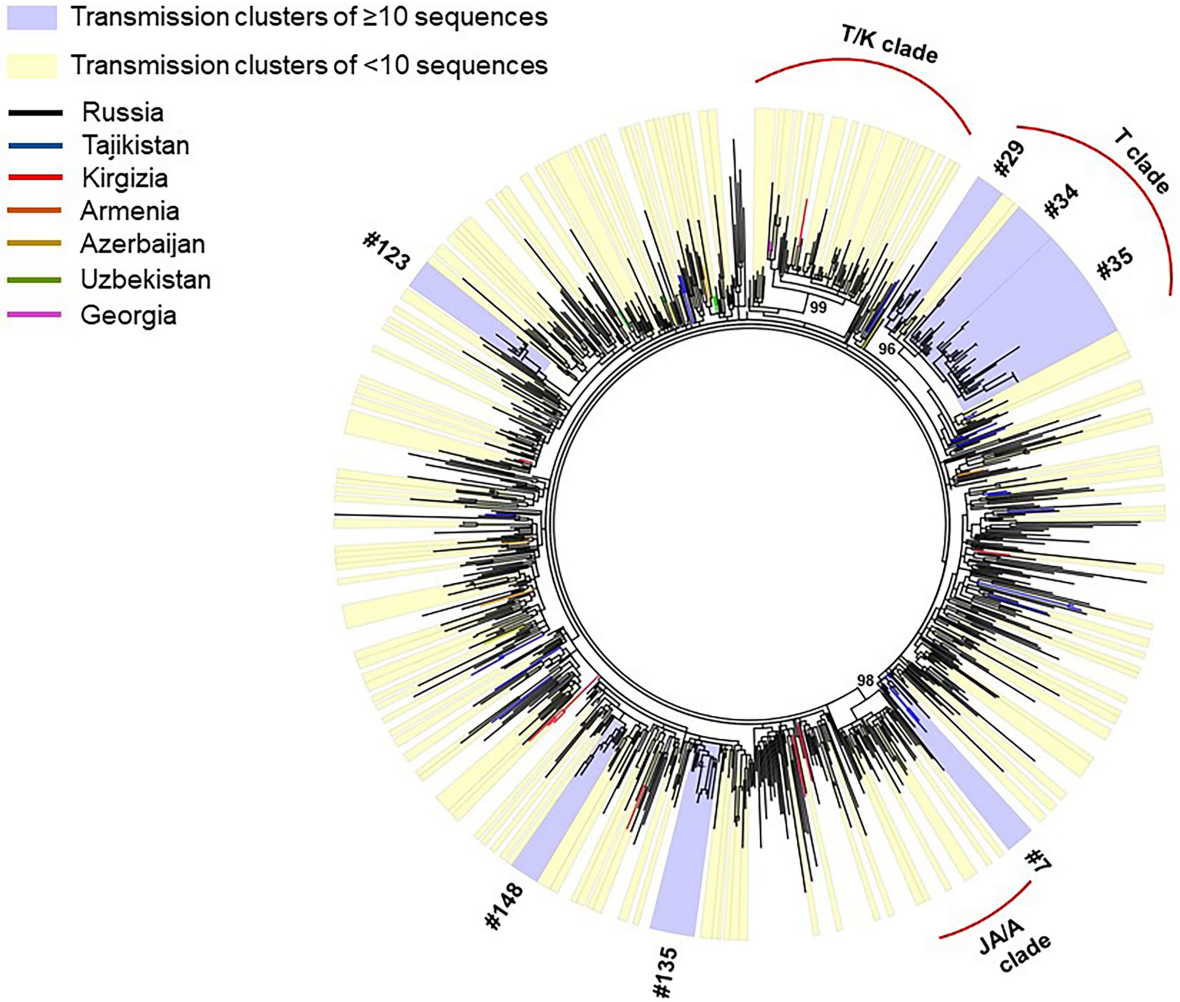
IQ-Tree of HIV-1 CRF63_02A 872 *pol* gene sequences. Tree branches color-codded according to the country of isolation (top left chart). Transmission clusters were identified using Cluster Picker v1.2.3 using a branch support value ≥90 and a maximum genetic distance threshold of 0.03. Transmission clusters of ≥10 sequences are colored blue; transmission clusters of <10 sequences are colored yellow.

Phylogenetic reconstruction was performed for 872 HIV-1 CRF63_02A6 *pol* gene sequences collected from 2009 to 2020 (Figure 2): 587 sequences were obtained by our research team and additional 285 sequences were retrieved form LANL HIV sequence database. Monophyletic structure of the CRF63_02A6 phylogenetic tree most likely indicates the initiation of the CRF63_02A6 sub-epidemic by a single ancestral virus. Predominant number (n = 832; 95.4%) of those sequences were from 25 regions of Russia; the remaining 40 (4.6%) sequences were from countries of Central Asia. A closer investigation ofHIV-1 CRF63_02A6 tree showed a total intermix of the sequences from different countries and regions across Russia supporting extensive virus distribution between those territories. Three well-supported (branch support value >95) location-specific monophyletic clades were detected: T/K clade was predominantly represented by CRF63_02A6 sequences from Tomsk and Krasnoyarsk regions (82% of the sequences within the clade), T clade was predominantly represented by sequences from Tomsk region (87% of the sequences within the clade), and JA/A clade was presented by sequences from Jewish Autonomous and Amur regions only. Those clades most likely represent separate introductions causing the super-spreading events of the virus. Recombination analysis revealed some evidence of recombination between CRF63_02A6 and sub-subtype A6 among sequences of the JA/A clade (n = 35, sequences dated 2018 and 2019); this could indicate the further local virus evolution.

Out of 872 sequences, 519 (59.5%) sequences were found in 157 transmission clusters (using thresholds of 0.03 of genetic distance and 90 of branch support) of size 2–38 sequences per cluster; Seven large (≥10 sequences) clusters were identified. The remaining 91 clusters were dyads, 38 clusters were triplets, 8 clusters were the size of four, 5 cluster were the size of five, 3 clusters were the size of six, two clusters were the size of seven, one cluster was the size of eight, and two clusters were the size of nine. The detailed information about large transmission clusters is provided in Table 1.

**Table 1:** Characteristics of the putative CRF63_02A6 transmission clusters.

Cluster ID	Cluster size^1^	Genetic distance	Geographic locations	Years^2^
7	10	0.029	TJ-Altai-NSK-Tomsk	2010–2017
29	10	0.027	Tomsk	2015–2016
34	15	0.03	Tomsk	2014–2016
35	38	0.03	Tomsk-KRS	2015–2018
123	10	0.029	NSK-Oryol	2016–2018
135	15	0.023	NSK-KRS-Omsk	2010–2017
148	10	0.024	NSK-Omsk	2011–2012

The table represents the size of transmission clusters, the maximum genetic distances between the sequences within the cluster, the region where the sequences were collected, and sequences collection dates. Genetic clusters were identified using Cluster Picker v1.2.3 with the maximum genetic distance of 0.03 and the branch support value ≥ 90.

1 Cluster size indicates the number of sequences in the transmission cluster.

2 The period when the samples within the cluster were collected.

TJ: Tajikistan; NSK: Novosibirsk region; KRS: Krasnoyarsk region.

### Spatiotemporal dynamics of HIV-1 CRF63_02A6

**Figure 3:**
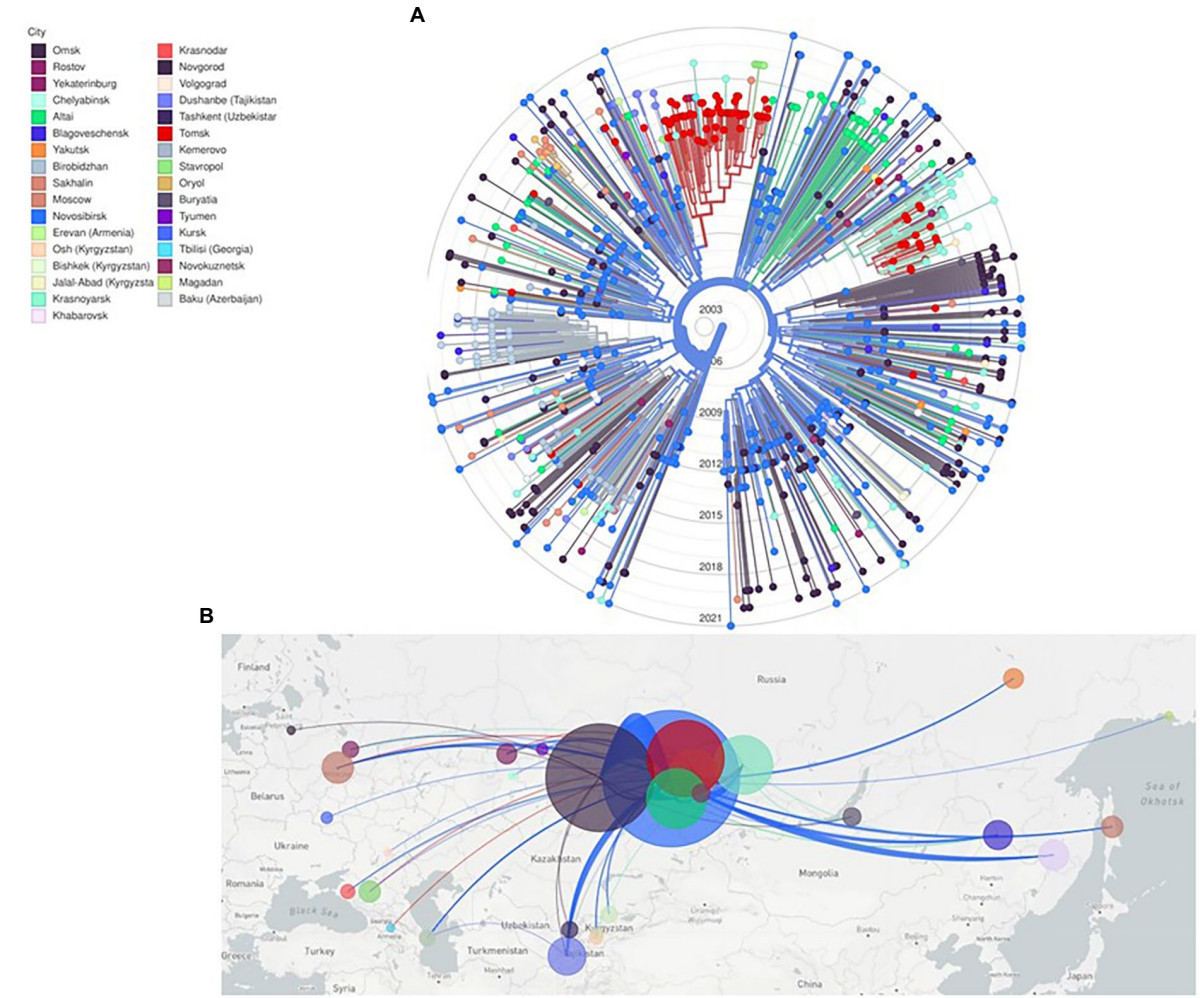
Nextstrain maximum likelihood analysis of HIV-1 CRF63_02A6 872 *pol* gene sequences. **(A)** RAx-ML tree was constructed using 832 sequences from Russia and 40 sequences from countries of Central Asia. Tree branches are colored according to the sampling city. **(B)** Geographical representation of CRF63_02A spatial dynamics. Lines between cities represent putative viral transitions; circle sizes are proportional to the square root of the number of sequences from the same location.

Phylogenetic and evolutionary analysis of CRF63_02A6 872 *pol* gene sequences using Nextstrain identified that CRF63_02A6 originated in Novosibirsk around February 2005 (confidence interval: December, 2004 – July, 2005; Figure 3). The virus was further transmitted across other Siberian territories (Omsk, Altai, Tomsk, Krasnoyarsk, and Kemerovo). By 2015, CRF63_02A6 was identified in 13 regions of Russia, and by 2020 the virus was also detected in other nine Russian regions and six countries of Central Asia.

### Phylodynamic reconstruction of HIV-1 CRF63_02A6

**Figure 4:**
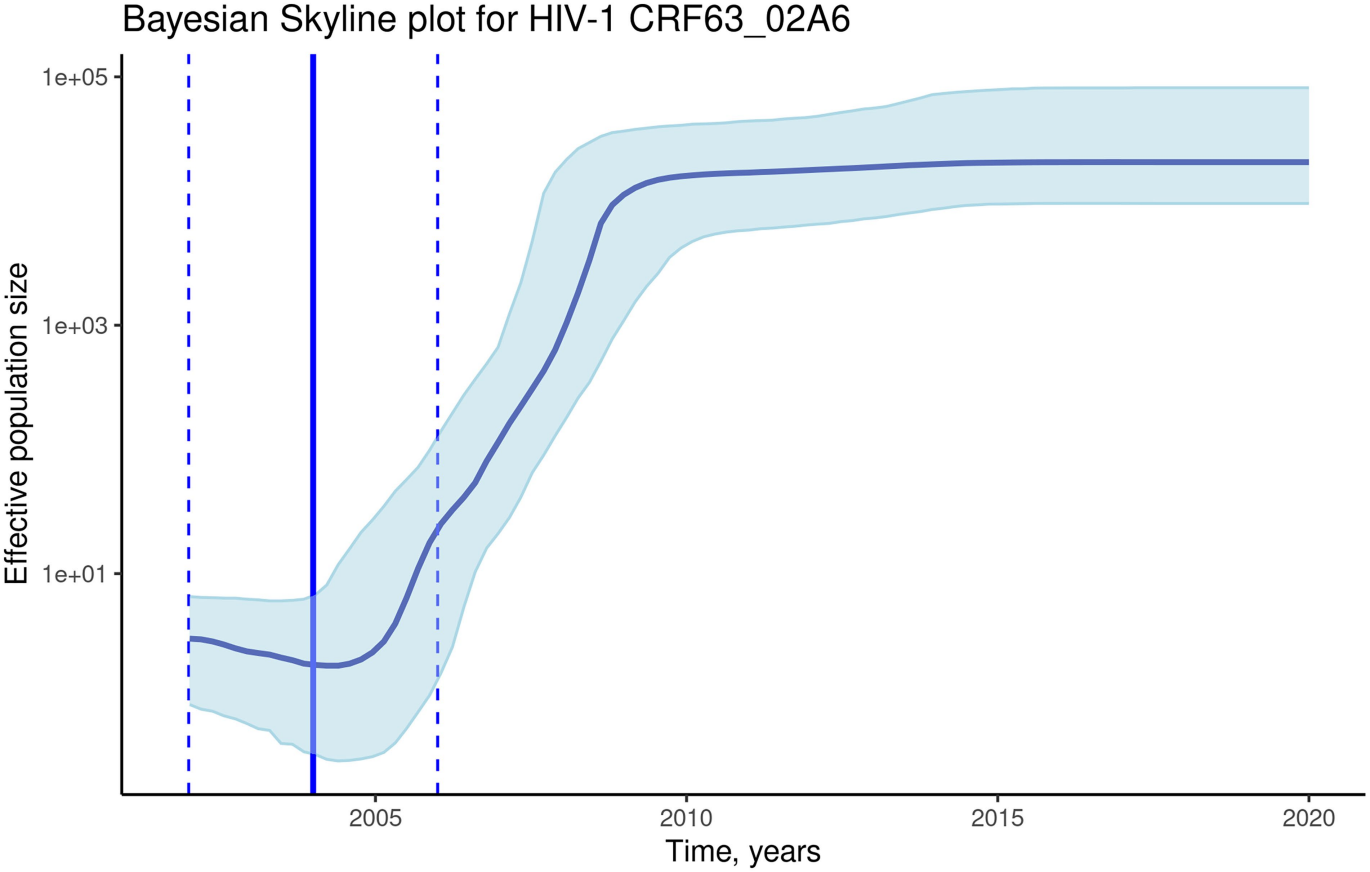
The Bayesian skyline plot of the 510 CRF63_02A6 *pol* gene sequences. The solid blue line indicates the median effective population size through time; the shaded blue area indicates the 95% highest posterior density (HPD) interval of the effective population size. The vertical solid black line indicates the median (2004) estimated tMRCA of the HIV-1 CRF63_02A6; the vertical dashed lines indicate the estimated tMRCA 95% HPD interval (2002–2006).

To investigate the evolutionary rate and population dynamics of the CRF63_02A6 using Bayesian phylogenetic inference, 510 *pol* gene sequences were included in the analysis. This number of sequences was defined using subsampling strategy described in Methods. The median estimated substitution rate was 2.3×10^−3^ substitutions/year (95% highest posterior density [HPD]: 1.9×10^−3^-2.7×10^−3^). The skyline plot shows an exponential growth of the ESS of the HIV-1 CRF63_02A6 from 2005 till 2010 followed by the stable phase (Figure 4). TMRCA was estimated as 2004 (95% highest posterior density: 2002–2006) using BEAST. Both results from the Nextstrain and Bayesian inference agreed and placed Novosibirsk as the most probable ancestral location of the HIV-1 CRF63_02A6 sub-epidemic. The tMRCA obtained by those two methods was also similar (2005 vs. 2004). Bayesian maximum clade credibility (MCC) tree and transmission history of the HIV-1 CRF63_02A6 constructed using SPREAD3 are presented in Supplementary Figure S3.

## Discussion

This report represents the characterization of the spatiotemporal dynamics of HIV-1 CRF63_02A6 sub-epidemic. This analysis includes 872 CRF63_02A6 *pol* gene sequences sampled in seven Eurasian countries. By the time the study was conducted, CRF63_02A6 sequences were sampled predominantly in Russia, along with the sporadic cases detected in six countries of Central Asia. The current analysis revealed that the onset date of HIV-1 CRF63_02A6 sub-epidemic was 2005 and the most probable origin location was Novosibirsk; the virus was subsequently transmitted to other regions of Russia and was also detected in Kyrgyzstan, Uzbekistan, Tajikistan, Azerbaijan, Armenia, and Georgia.

The HIV-1 genetic diversity in Russia is broad and characterized by the region-specific sub-epidemics. The first HIV-1 infection outbreak was registered in 1988–1989 among nosocomially infected children in the South of Russia and was caused by the HIV-1 subtype G (Thomson and Najera, 2007; Serova et al., 2018), but this subtype is currently rarely detected in the country. In the mid-1990s, the multiple HIV-1 outbreaks among PWID were detected in Central Russia (Kazennova et al., 2014) and gave rise to an extensive expansion of the HIV-1 epidemic in the country caused by the sub-subtype A6. In the following years, the virus was detected in different Russian regions and neighboring countries (Thomson et al., 2009) and currently predominates across all FSU countries. Subtype B was identified among MSM population and presently accounts for up to 2–5% of all HIV-1 cases (Gashnikova et al., 2015; Pasechnik and Blokh, 2018; Lebedev et al., 2019). Recombinant form of subtype B and sub-subtype A6 - CRF03_AB caused several outbreaks among PWID in the Northwestern Russia (Lebedev et al., 2020) and predominates alongside with the sub-subtype A6 (Ozhmegova et al., 2020). HIV-1 genetic diversity in the Russian Far East is different from the rest of the country. Besides the predominant sub-subtype A6, subtype B and subtype C were found to account for 25% and 10% of the HIV-1 cases, respectively (Kazennova et al., 2008).

Up to mid-2000s, sub-subtype A6 predominated in Siberia. But since 2008, a rapid spread of novel HIV-1 genetic variant was detected in Novosibirsk (Gashnikova et al., 2011). Detailed full genome analysis identified this genetic variant as a recombinant of sub-subtype A6 and CRF02_AG_FSU_; phylogenetic analysis revealed that the new recombinant form (subsequently named as CRF63_02A6) was genetically related to the CRF02_AG_FSU_ from Uzbekistan (Baryshev et al., 2012). A retrospective molecular-epidemiological analysis revealed that some of the PWLH in Novosibirsk were HIV-infected by this recombinant form back in 2004–2006, when the local outbreaks among PWID were registered (Gashnikova et al., 2011). The HIV-1 CRF63_02A6 sub-epidemic distribution in Russia continued and the number of CRF63_02A6 cases started to increase in other regions (Safina et al., 2022). CRF63_02A6 was also detected in the countries of Central Asia, however those cases are sporadic and most likely represent cross-border transmission events (Sivay et al., 2021). In the current study, a large-scale phylogenetic analysis of over 2,500 *pol* gene sequences showed that CRF63_02A6 forms a well-supported distinct monophyletic clade, which most likely indicates the initiation of the CRF63_02A6 epidemic by a single ancestral virus. By now, CRF63_02A6 was detected in over 20 Russian territories and six countries of Central Asia. The current phylogenetic analysis of the CRF63_02A6 revealed three distinct region-specific clades, most likely indicating the local super-spreading events.

Earlier studies (Kostaki et al., 2018) on demographic history and phylodynamic analysis of the CRF02_AG/CRF63_02A6 combined CRF63_02A6 and CRF02_AG_FSU_ lineages. The study identified the origin of the CRF63_02A6 in Kazakhstan and Uzbekistan in 1996; two Russian subclades dated 2003 and 2007 were detected. By the time that study was conducted, a very limited number (n = 136) of the CRF63_02A6 sequences were available. Our study and the study of the global dissemination of the CRF02_AG (Mir et al., 2016) showed that CRF63_02A6 and CRF02_AG_FSU_ represent two distinct HIV-1 genetic variants. Given the results of our previous study and earlier report by Baryshev et al. (2012), CRF63_02A6 most likely descended from CRF02_AG_FSU_ and is characterized by a significant genome change compared to CRF02_AG_FSU_. Both of those CRFs have different geographic characteristics. Thus, CRF_02AG_FSU_ is endemic in Central Asia (Sivay et al., 2021), while CRF63_02A6 predominantly circulates in Russia. Current report includes all the available HIV-1 CRF63_02A6 and CRF02_AG_FSU_ sequences published in the LANL HIV sequence database from the Eurasian continent (Supplementary Figure S1). Our analysis shows that all the *pol* gene sequences from Kazakhstan available in LANL HIV Sequences Database belong to CRF02_AG_FSU_. Our analysis also finds only a few CRF63_02A6 sequences from Uzbekistan dated 2015; most of the HIV-1 sequences from Uzbekistan are CRF02_AG_FSU_.

HIV-1 epidemic in countries of Central Asia was driven by the drug trafficking through the so called “Northern route” from Afghanistan to Russia through Tajikistan, Uzbekistan, Kirgizia, and Kazakhstan (Vorobyev, 2014; Merz, 2018). Since earlier-2000s, the rapid increase of drug use was registered in Russia, and from 2002 to 2014 Siberia was leading by the number of PWID in the country (Andreev and Kokunova, 2017). Also, an intensive labor migration from Central Asian countries to Novosibirsk was registered. Those factors could create favorable conditions for HIV-1 transmission. According to Andreev and Kokunova (2017), a high level of drug use was found among HIV-infected illegal labor migrants. Rapid CRF63_02A6 sub-epidemic expansion in Siberia could also be linked to the high synthetic drug use starting in 2009. Since 2011, the decline in opioid drugs use together with an increase in synthetic drugs use were registered (Vorobyev, 2014; Andreev and Kokunova, 2017). Synthetic drugs induce both stimulant and sexual effects promoting high-risk sexual behavior (Luo et al., 2018). Active synthetic drug use could contribute to dissemination of distinct CRF63_02A6 lineages resulting to a fold-increase of new HIV-1 cases in Siberian regions (Gashnikova et al., 2015, 2017). The well-supported region-specific monophyletic CRF63_02A6 clades identified in the current study most likely indicate these super-spreading events (Figure 2).

Since 2005, the number of new HIV-1 cases in Novosibirsk has started to grow exponentially. The largest increase (from 19.6% in 2003 to 57% in 2007) in new HIV-1 cases in the region was recorded among prisoners (Novosibirsk Region Center on AIDS, 2014).^3^ Most likely such increase was driven by the high-risk behavior, such as intravenous drug use (IDU). Most people who entered the Russian penal system in mid-2000s were PWID (Severson, 2011). The penal system had transformed into a unique risk environment for PWID and HIV-1 transmission due to the lack of harm reduction programs. An earlier study among prisoners in Russia showed that almost a half of them had a history of drug injection; that 20% of prisoners had used drugs while imprisoned, 64% of those individuals had injected drugs with previously used injecting equipment, and that 13.5% had started injecting drugs while imprisoned (Jürgens and Betteridge, 2005). Several HIV-1 outbreaks associated with IDU were registered among prisoners in Russia (Bobrik et al., 2005; Severson, 2011). We admit that the emergence of the HIV-1 CRF63_02A6 could have happened within one of the prisons in the Novosibirsk.

In Russia, the guidelines for laboratory testing of HIV-1 infection requires a fourth-generation HIV-EIA test (4th Gen), which detects both antibody and antigen with the following confirmatory Western Blot testing and/or HIV-1 RNA testing (Ministry of Health of Russian Federation, 2020).^4^ However, HIV-1 RNA testing is not routinely used and is generally provided to those individuals who have previously confirmed HIV-1 infection, as well as children and pregnant women. Patients with the acute HIV-1 infection can be misdiagnosed during the HIV-1 seronegative window period and can easily transmit the virus. Earlier report described clinical characteristics of patients with acute HIV-1 infection in Novosibirsk. Most of the patients (76%) had CRF63_02A6 infection and 19.4% had sub-subtype A6 infection. Statistically significant differences were found between clinical manifestation in patients with the HIV-1 CRF63_02A6 and sub-subtype A6 infection. Thus, CRF63_02A6 infection is characterized by more severe primary symptoms, such as prolonged fever, generalized lymphadenopathy, higher (>10^7^) viral load, and lower CD4 cell counts compared to patients with the sub-subtype A6 infection (Ulyanova et al., 2019). These data indicate a faster disease progression and rapid virus transmission among patients with the CRF63_02A6 compared to sub-subtype A6 infection. Symptoms of acute HIV-infection are nonspecific, and the infection often can be misdiagnosed, especially within the ongoing COVID-19 pandemic and COVID-19 mass vaccination. Beginning in 2000, our research team initiated HIV-1 surveillance program in Novosibirsk, which was also launched in other regions of Siberia around 2010. Our studies detected the displacement of sub-subtype A6 by CRF63_02A6, and started in 2009 CRF63_02A6 has been detected in over 80% of new HIV-1 cases in Siberia. Also, various URFs of sub-subtype A6 and CRF63_02A6 has been persistently detected (Gashnikova et al., 2015, 2017; Ulyanova et al., 2019).

Current analysis identified a misclassification of CRF63_02A6 as CRF02_AG by widely used HIV-1 automated subtyping tools. HIV-1 has a high recombination rate promoting viral genetic diversification and escape from the host immune system (Levy et al., 2004). New CRFs are constantly detecting and 118 of them have been described by now (Los Alamos National Laboratory HIV Sequence Database, 2022b).^5^ Proper identification of HIV-1 subtypes is challenging due to the frequent recombination. Molecular phylogeny remains the most reliable HIV-1 subtyping approach (Fabeni et al., 2017), however this method requires the data manipulation and interpretation skills. In the clinical settings, HIV-1 subtyping is routinely based on fast and simple HIV-1 automated subtyping tools which have some limitations compared to the phylogenetic approach. Automated subtyping tools often have a very limited number of CRFs in the reference dataset. Most of the automated HIV-1 subtyping algorithms are designed mainly for the most prevalent viral subtypes such as B and C, and CRFs such as CRF01_AE and CRF02_AG (Fabeni et al., 2017). Also, those tools are not frequently updated. In the current report, we used two automated HIV-1 subtyping tools, Stanford and COMET, and maximum likelihood phylogenetic approach (combined with 2020 LANL HIV-1 subtype reference sequences) to identify CRF63_02A6 sequences. The HIV-1 CRFs were assigned based on high phylogenetic relatedness of the study sequences with the corresponding HIV-1 subtype reference sequences. HIV-1 subtyping results of CRF63_02A6 sequences obtained by Stanford and COMET automated subtyping tools were concordant with the phylogenetic analysis results in approximately 75 and 79% instances, respectively. Moreover, a third of CRF02_AG_FSU_ sequences were classified as CRF63_02A6 by COMET. Our data highlight a rather limited performance of Stanford and COMET subtyping tools in CRF63_02A6 assignment compared to the phylogenetic approach. Although the automated HIV-1 subtyping tools are widely used in clinical settings, the reference datasets of these tools need to be updated to improve reliability of the HIV-1 subtyping results.

This report provides a comprehensive analysis on the origin and dissemination of the recent HIV-1 CRF63_02A sub-epidemic in Russia and countries of Central Asia. The study describes the onset of the CRF63_02A6 in Siberia in the mid-2000s and its following spreading across Russia and the neighboring countries. The CRF63_02A6 distribution simultaneously occurred with the use of injectable and synthetic drugs. Since 2016, the HIV-1 heterosexual transmission in Siberia has prevailed over PWID, however HIV-1 incidence rate among PWID remains high. A high prevalence (from 2 to 30% across the regions) of HIV-1 URFs indicates a substantial level of co-infection with different HIV-1 subtypes. Further surveillance studies of the HIV-1 CRF63_02A6 are needed due to the continuing rapid distribution and high rate of recombination between CRF63_02A6 and other HIV-1 subtypes/CRFs. Studies of epidemiological characteristics and their relationships with the new emerging HIV-1 genetic variants are urgently needed. Also, the introduction of HIV-1 prevention and treatment programs are required to implement further HIV-1 transmission reduction strategies. This study also emphasizes importance of updating of the reference datasets of the widely used automated HIV-1 subtyping tools to provide reliable HIV-1 subtyping results.

## Data availability statement

The data presented in this study are deposited in the GenBank repository, accession numbers are listed in Supplementary Data.

## Ethics statement

The studies involving human participants were reviewed and approved by Local Ethical Committee of the State Budgetary Healthcare Institution of Novosibirsk Region “City Infectious Clinical Hospital #1”. Written informed consent to participate in this study was provided by the participants’ legal guardian/next of kin.

## Author contributions

MS, AT, and NG planned and designed the study. LM, IO, AN, MG, DZ, VE, TN, and VI performed HIV genotyping and collected sequences. MS performed the analysis of the epidemiological data, performed the phylogenetic and phylodynamic analysis, produced the illustrations, and wrote the manuscript. NG supervised the project and edited the manuscript. DK, LP, SS, TO, SY, ON, AC, and TI collected the epidemiological data and assisted with samples collection at the local study clinics. All authors contributed to the article and approved the submitted version.

## Funding

This study was supported by the State Research and Development Program #6–21 State Research Center of Virology and Biotechnology “Vector.”

## Conflict of interest

The authors declare that the research was conducted in the absence of any commercial or financial relationships that could be construed as a potential conflict of interest.

## Publisher’s note

All claims expressed in this article are solely those of the authors and do not necessarily represent those of their affiliated organizations, or those of the publisher, the editors and the reviewers. Any product that may be evaluated in this article, or claim that may be made by its manufacturer, is not guaranteed or endorsed by the publisher.

## Footnotes

^1^ http://www.hivrussia.info/wp-content/uploads/2022/03/Spravka-VICH-v-Rossii-na-31.12.2021-g.pdf

^2^ http://www.hiv.lanl.gov/

^3^ https://oblmed.nsk.ru/upload/files/hiv.pdf

^4^ http://rushiv.ru/category/docs/national-recs/kr-20/

^5^ https://www.hiv.lanl.gov/content/sequence/HIV/CRFs/CRFs.html
