# Supplementary figures and images for: Spatiotemporal dynamics of HIV-1 CRF63_02A6 sub-epidemic
Source: Front Microbiol. 2022 Aug 31;13:946787. doi: 10.3389/fmicb.2022.946787 (PMC9470837; doi:10.3389/fmicb.2022.946787)

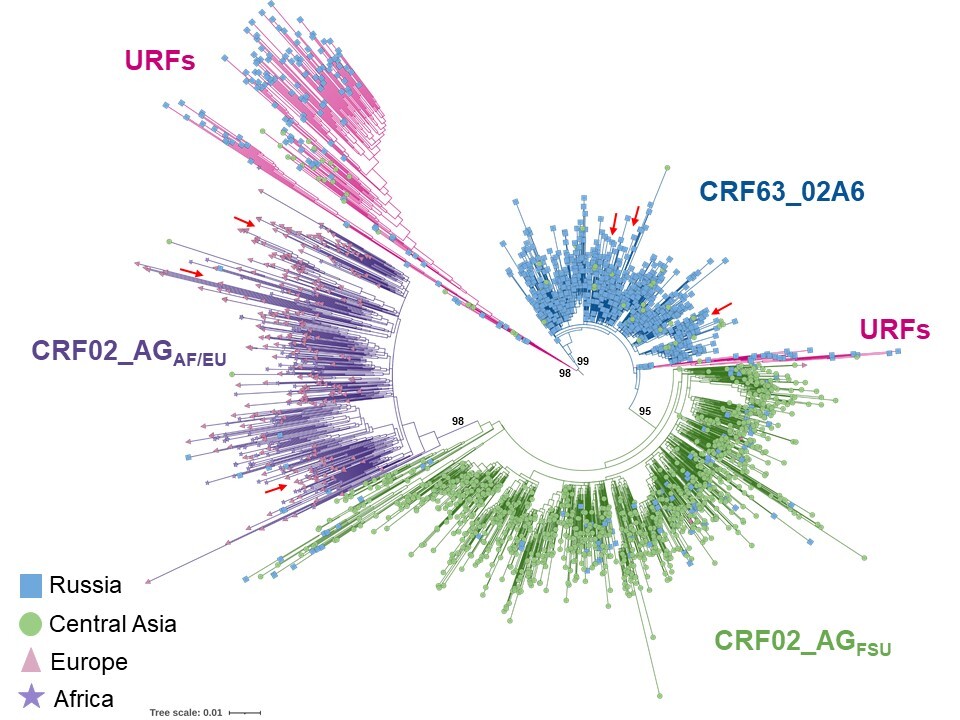

Supplement: Supplementary file 1 [file Image_1.JPEG]

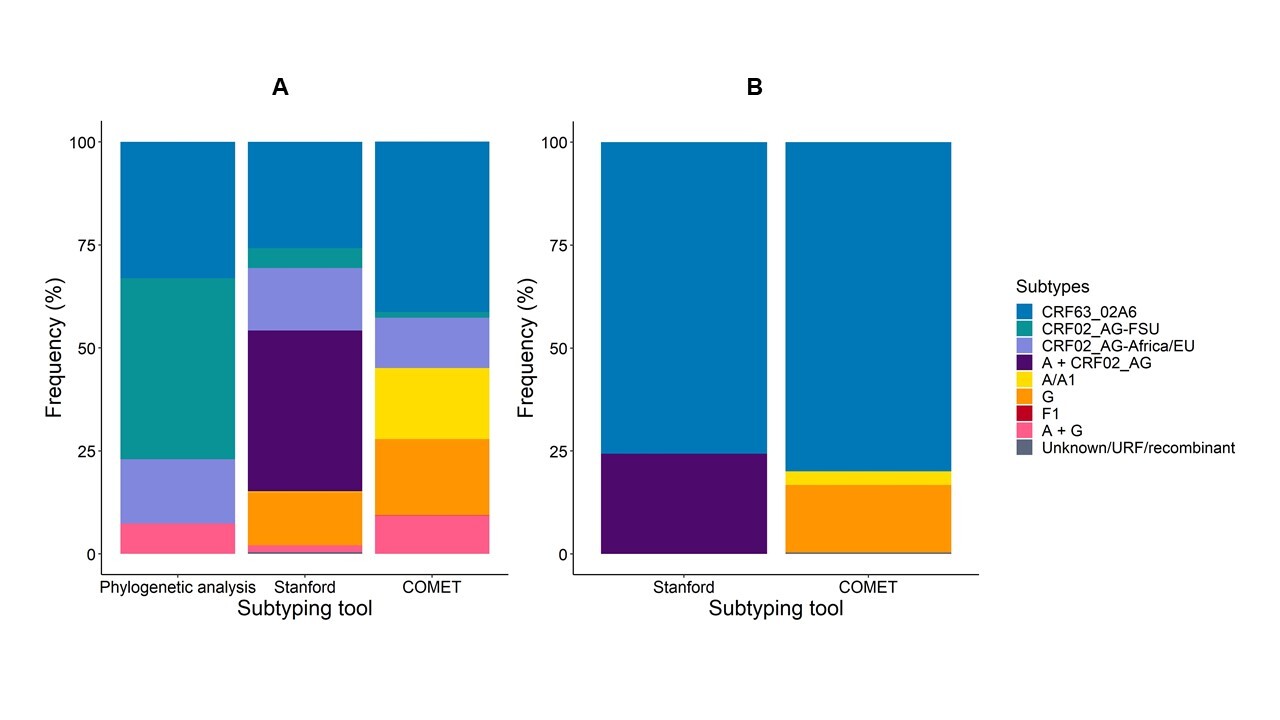

Supplement: Supplementary file 2 [file Image_2.JPEG]

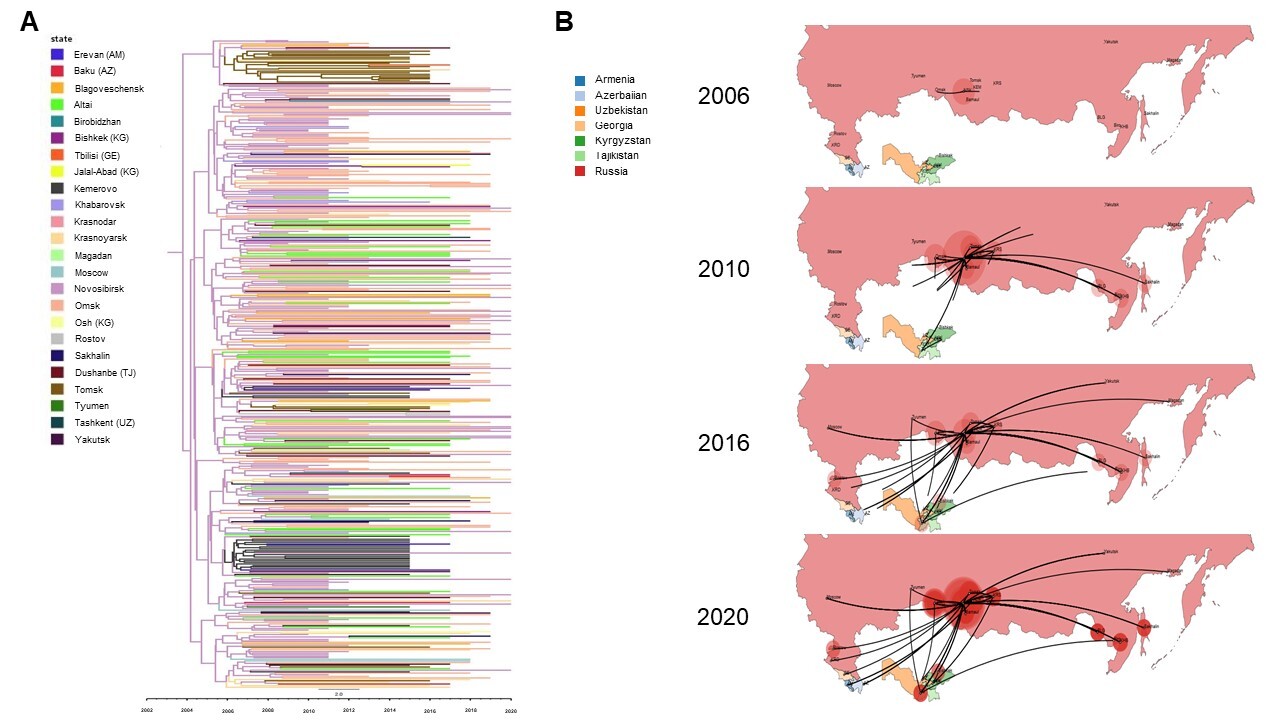

Supplement: Supplementary file 3 [file Image_3.JPEG]
