# Supplementary material for: Spatiotemporal dynamics of HIV-1 CRF63_02A6 sub-epidemic
Source: Front Microbiol. 2022 Aug 31;13:946787. doi: 10.3389/fmicb.2022.946787 (PMC9470837; doi:10.3389/fmicb.2022.946787)
Supplement: Supplementary file 4 [file Data_Sheet_1.PDF]

MW30361619
MW30361419
MW30361218
MW30361318
MW30361719
MW30361519
MG79901417
MG79911617
MN64929019
MN64928118
MN64929119
MN64928218
MK00256717
MW22921218
MN64929219
MN64928318
MN64929319
MW22921318
MN64929419
MK00256817
MN64928418
MN64928518
MN64929519
JX29027109
HQ12997809
HQ12998009
HQ12998109
HQ12998209
JX50070109
KJ87037011
MK00256917
MN64928618
MN64929619
MK00258618
MK00258718
MN64933919
MK00258818
HQ12998310
HQ12998410
HQ12998510
HQ12998610
HQ13000510
HQ13000610
HQ12998810
JX29024910
JX50069911
JX29025010
JX29025110

JX29025210
JX29025310
JX29025410
JX29025510
JX29025610
JX50070210
JX29025710
JX29025810
JX29025910
JX29026010
JN23035310
JX29026210
JX29026310
JX50070010
JX29026410
JX29026510
JX29026610
JX29026710
JX29026810
JX50070410
JX29026910
MN64934019
MN64934119
MN64934219
MK00258918
MW22922020
MK00259018
KF37477811
KF37477911
KF37478011
KF37478111
KF37478211
KF37478311
KF37478411
KF37478511
KF37478611
KF37478711
KF37478811
KF37478911
KF37479011
KF37479111
KF37479211
KF37479311
KF37479411
KF37479511
KF37479611
KF37479711
KF37479811

KF37479911
KF37480011
KF37480111
KF37480211
KF37480311
KF37480411
KF37480511
KF37480711
KF37480811
KF37480911
KF37481011
KF37481111
KF37481211
KF37481311
KF37481411
KF37481511
KF37481611
KF37481711
KF37481811
KF37481911
KF37482011
KF37482111
KF37482211
KF37482311
KF37482411
KF37482511
KF37482611
KF37482711
KF37482811
JX50069811
KF37472511
KF37472611
KF37472711
KF37472811
KF37472911
KF37473011
KF37473111
KF37473211
KF37473311
KF37473411
KF37473511
KF37473611
KF37473711
KF37473811
KF37473911
KF37474111
KF37474211
KF37474311

KF37474411
KF37474511
KF37474611
KF37474711
KF37474811
KF37474911
KF37475011
KF37475111
KF37475211
KF37475311
KF37475411
KF37475511
KF37475611
KF37475711
KF37475811
KF37475911
KF37476011
KF37476111
KF37476211
KF37476311
KF37476411
KF37476511
KF37476611
KF37476711
KF37476811
KF37476911
KF37477011
KF37477111
KF37477211
KF37477311
KF37477411
KF37477511
KF37477611
KF37477711
MW22921418
MN64928718
MN64929719
MK00259118
MK00259218
KF37482912
KF37483012
KF37483112
KF37483212
KF37483312
KF37483412
KF37483512
JX50070512
KF37483612

KF37483712
KF37483812
KF37483912
KF37484012
KF37484112
KF37484212
KF37484312
KF37484412
KF37484512
KF37484612
KF37484712
KF37471012
KF37471112
KF37471212
KF37471312
KF37471412
KF37471512
KF37471612
KF37471712
KF37471812
KF37471912
KF37472012
KF37472112
KF37472212
KF37472312
KF37472412
MK00257017
MN64929819
MW22922120
KF37484813
KF37484913
KF37485013
MK00257117
MW22922220
MN64930019
MN64930119
MW22922320
MW22924120
MG21153814
MG21153914
MG21154014
MG21154114
MG21154214
MG21154314
MG21154414
MG21154514
MK00257217
MG21154615

MG21154815
MG21155015
MG21155115
MG21155215
MG21155315
MG21155415
MG21155515
MG21155815
MG21155915
MG21156015
MG21156115
MG21156215
MG21156315
MG21156515
MG21156615
MG21156715
MG21156815
MG21156915
MG21157015
MG21157115
MG21157215
MG21157315
MG21157415
MG21157515
MG21157615
MT10179916
MT10180016
MT10180216
MT10180316
MT10180416
MT10180616
MT10180816
MT10180916
MT81109616
MT10181016
MT81109716
MT10181116
MT10181216
MT10181316
MT10181416
MT10181516
MT10181616
MT10181716
MT10181816
MT10181916
MT10182016
MT10182116
MT10182216

MT10182316
MT10182616
MT10182716
MT10182816
MT81109916
MT10182916
MT10183216
MT10183316
MT10183416
MG21157716
MG21157816
MG21157916
MG21158016
MG21158116
MG21158216
MG21158316
MG21158416
MG21158516
MG21158616
MG21158716
MG21158816
MG21158916
MG21159016
MG21159116
MG21159216
MG21159316
MG21165616
MG21159416
MG21159516
MG21159616
MG21159716
MG21165716
MG21159816
MG21159916
MG21160016
MG21160116
MG21160216
MG21160316
MG21160416
MG21160516
MG21160616
MG21160716
MG21160816
MG21160916
MG21161016
MG21161116
MG21161216
MG21161316

MG21161416
MG21161516
MG21161616
MG21161716
MG21161816
MG21161916
MG21162016
MG21162116
MG21162216
MG21162316
MG21162416
MG21162516
MG21162616
MG21162716
MG21162816
MG21162916
MG21166116
MK00257317
MT81110218
MT81110318
MT81110418
MT81110618
MT81111218
MT81111418
MK00257417
MN64928818
MN64930219
MW22921518
MN64928918
MN64930319
MW22921619
MN64930519
MN64930619
MN64930819
MN64930919
MW22921719
MN64931019
MW22921819
MN64931219
MN64931319
MN64931419
MK00257517
MK00257617
MN64931619
MN64931719
MN64931819
MW22921919
MN64932019

MN64932119
MK00257717
MN64932219
MN64932319
MK00257817
MN64932419
MN64932519
MN64932619
MK00257917
MN64932719
MN64932819
MN64932919
MN64933019
MN64933119
MN64933219
MN64933319
MK00258118
MN64933419
MK00258218
MN64933519
MK00258318
MN64933619
MK00258418
MK00258518
MN64933819
KX57435715
KX57435815
KX57435915
KX57436015
KX57436115
KX57436215
KX57436315
KX57436415
KX57436515
KX57436615
KX57436715
KX57436815
KX57436915
KX57437015
KX57437115
KX57437215
KX57437315
KX57437415
KX57437515
KX57437615
KX57437715
KX57437815
KX57437915

KX57438015
KX57438115
KX57438215
KX57438315
KX57438415
KX57438515
KX57438715
KX57438615
KX57438815
KX57438915
KX57439015
KX57439115
MK98415917
MK98416015
KX64018115
KX64018215
MK24559317
MK24559417
MH54330317
MH54330417
MK24559817
MH54330517
MK24559517
MK24559617
MH54330617
MH54330717
MH54330817
MH54330917
MH54331017
MH54331117
MH54329917
MK24576917
MH54330017
MK24577017
MK24577117
MH54330117
MK24559717
MH54330217
ON528587-ON528679
