# Supplementary material for: Spatiotemporal dynamics of HIV-1 CRF63_02A6 sub-epidemic
Source: Front Microbiol. 2022 Aug 31;13:946787. doi: 10.3389/fmicb.2022.946787 (PMC9470837; doi:10.3389/fmicb.2022.946787)
Supplement: Supplementary file 5 [file Data_Sheet_2.DOCX]

Supplementary Material

# Supplementary Data. Accession numbers of HIV-1 *pol* gene sequences analyzed in the study.

# Supplementary Figures Legends

**Supplementary Figure 1.** IQ-Tree phylogenetic tree of the HIV-1 2,637 *pol* gene sequences. Dataset includes CRF63_02A6 (n=872), CRF02_AG_FSU_ (n=1157), CRF02_AG_AF/EU_ (n=412), and URF (n=196) sequences. Red arrows indicate HIV-1 CRF63_02A6 and CRF02_AG references. Phylogenetic tree was visualized in iTOL v6.

**Supplementary Figure 2.** Comparative analysis for HIV-1 CRF63_02A6 *pol* gene sequences subtyping results. Subtyping results were obtained using phylogenetic analysis (using IQ-Tree including 2020 LANL HIV-1 reference sequence list) and two automated HIV-1 subtyping tools Stanford HIVdb Program and COMET. A. Subtyping results of 2,637 *pol* gene sequences include all CRF02_AG and CRF63_02A6 and sequences of CRF63_01A, CRF02A6, CRF02A1, and unknown subtypes available in the LANL HIV-1 sequence database from FSU countries. Additional 412 CRF02_AG sequences from African and European countries were also included. B. Stanford HIVdb Program and COMET subtyping results of sequences classified as CRF63_02A6 (n=872) by phylogenetic analysis.

**Supplementary Figure 3.** Phylogeographic analysis of the 510 *pol* gene sequences of the HIV-1 CRF63_02A6. A. Bayesian maximum clade credibility (MCC) time scaled discrete tree. The estimated tMRCA was 2004 (95% HPD: 2002-2006). B. Geographical representation of the transmission history of the HIV-1 CRF63_02A6 constructed using SPREAD3 v0.9.7.1. Novosibirsk had the highest posterior probability (pp=1) for the root of the tree.
